# Carbon for nutrient exchange between arbuscular mycorrhizal fungi and wheat varies according to cultivar and changes in atmospheric carbon dioxide concentration

**DOI:** 10.1111/gcb.14851

**Published:** 2019-10-23

**Authors:** Tom J. Thirkell, Daria Pastok, Katie J. Field

**Affiliations:** ^1^ Centre for Plant Sciences School of Biology Faculty of Biological Sciences University of Leeds Leeds UK

**Keywords:** arbuscular mycorrhizal fungi, carbon, climate change, CO_2_, nitrogen, phosphorus, sustainable agriculture, wheat

## Abstract

Arbuscular mycorrhizal fungi (AMF) form symbioses with most crops, potentially improving their nutrient assimilation and growth. The effects of cultivar and atmospheric CO_2_ concentration ([CO_2_]) on wheat–AMF carbon‐for‐nutrient exchange remain critical knowledge gaps in the exploitation of AMF for future sustainable agricultural practices within the context of global climate change. We used stable and radioisotope tracers (^15^N, ^33^P, ^14^C) to quantify AMF‐mediated nutrient uptake and fungal acquisition of plant carbon in three wheat (*Triticum aestivum* L.) cultivars. We grew plants under current ambient (440 ppm) and projected future atmospheric CO_2_ concentrations (800 ppm). We found significant ^15^N transfer from fungus to plant in all cultivars, and cultivar‐specific differences in total N content. There was a trend for reduced N uptake under elevated atmospheric [CO_2_]. Similarly, ^33^P uptake via AMF was affected by cultivar and atmospheric [CO_2_]. Total P uptake varied significantly among wheat cultivars and was greater at the future than current atmospheric [CO_2_]. We found limited evidence of cultivar or atmospheric [CO_2_] effects on plant‐fixed carbon transfer to the mycorrhizal fungi. Our results suggest that AMF will continue to provide a route for nutrient uptake by wheat in the future, despite predicted rises in atmospheric [CO_2_]. Consideration should therefore be paid to cultivar‐specific AMF receptivity and function in the development of climate smart germplasm for the future.

## Graphical abstract

Arbuscular mycorrhizal fungi (AMF) form symbioses with most crops, potentially improving their nutrient assimilation with possible applications in future sustainable agriculture. However, the effects of cultivar and increasing CO_2_ on wheat mycorrhizal function remain unknown. Using isotope tracers, we tracked mycorrhizal function in three wheat cultivars across two CO_2_ scenarios. Our results suggest AMF could provide a route of nutrient uptake to wheat in the future, despite predicted rises in atmospheric CO_2_. Consideration should be paid to cultivar‐specific AMF receptivity and function in the development of climate smart future crops.

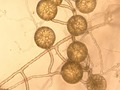

## 1 INTRODUCTION

The agricultural ‘green revolution’ of the 1950s brought dramatic increases in worldwide crop productivity, driven largely by the development and application of novel pesticides and fertilizers, coupled to advances in plant breeding. Such crop improvements are exemplified by the long‐term increase in UK wheat yields since the 1950s (Mackay et al., 2011). More recently, wheat yields have started to decline despite increasing application of nitrogen‐ and phosphorus‐based fertilizers—a widespread trend observed across many other key crop species across the globe (Grassini, Eskridge, & Cassman, 2013; Ray, Ramankutty, Mueller, West, & Foley, 2012). An ever‐increasing human population (Gerland et al., 2014), depletion of natural resources such as rock phosphate (Cordell, Drangert, & White, 2009) and rising energy prices are making fertilizer and pesticide production unsustainable. In the context of global climate change, future food security is far from assured (Godfray et al., 2010).

In recent years, there has been increasing agronomic interest in exploiting the symbiotic associations formed between crop plants and arbuscular mycorrhizal fungi (AMF; Chen, Arato, Borghi, Nouri, & Reinhardt, 2018; Sosa‐Hernandez, Leifheit, Ingraffia, & Rillig, 2019; Thirkell, Charters, Elliott, Sait, & Field, 2017). The roots of around 75% of all vascular plant species, including many cereals (Smith & Smith, 2011) form associations with the obligately biotrophic fungi of the subphyllum Glomeromycotina (Brundrett & Tedersoo, 2018; Spatafora et al., 2016; van der Heijden, Martin, Selosse, & Sanders, 2015). Host plants may allocate up to 20% of recently‐fixed carbon (C) to their AMF symbionts (Bago, Pfeffer, & Shachar‐Hill, 2000; Douds, Pfeffer, & Shachar‐Hill, 2000; Soudzilovskaia et al., 2015). On a global scale, such transfer of carbohydrates and fatty acids (Keymer et al., 2017; Luginbuehl et al., 2017) from plants to fungal partners comprises up to 5 billion tons of C annually (Bago et al., 2000), representing an important input to soil carbon stocks. In return, AMF may facilitate the acquisition of up to 80% of plant phosphorus (P; Bucher, 2007; Sawers et al., 2017; Smith, Smith, & Jakobsen, 2004), in addition to potentially making contributions towards plant nitrogen (N; Hodge, Campbell, & Fitter, 2001; Leigh, Hodge, & Fitter, 2009; Thirkell, Cameron, & Hodge, 2016) and micronutrient demand (Smith & Read, 2008). Associating with AMF may confer further benefits on host plants beyond improving access to soil nutrients, such as improving plant growth, water uptake (Ruiz‐Lozano et al., 2016) and priming of host plant defence responses (Cameron, Neal, Wees, & Ton, 2013), leading to increased tolerance and/or resistance to pests and diseases (Berdeni et al., 2018; Jung, Martinez‐Medina, Lopez‐Raez, & Pozo, 2012).

Taking consideration of AMF in widescale agricultural management decisions requires changes in current practice, although it has been argued that sufficient data corroborating the nutritional benefit of AMF in agricultural crops to warrant these shifts are currently lacking (Rillig et al., 2019; Ryan & Graham, 2018). A prevailing assertion is that cereals are generally negatively or neutrally affected by AMF colonization (Rillig et al., 2019; Smith & Smith, 2011); the fungi are assumed to offer little nutritional benefit to plants selectively bred for fine and dense root architecture optimized for nutrient‐acquisition efficiency, especially under high‐nutrient environments (Smith & Smith, 2011; Wen et al., 2019; Zheng et al., 2018). Despite two meta‐analyses suggesting an overall benefit of AMF to crop nutrient uptake and grain yield (Lekberg & Koide, 2005; Zhang, Lehmann, Zheng, You, & Rillig, 2019), a sceptical view remains in the literature with regard to the utility of AMF in modern and future agriculture (e.g. Ryan & Graham, 2018).

The functional response of plants to AMF colonization is highly diverse (Hoeksema et al., 2010) in terms of both inter‐ and intraspecificity (Johnson, Martin, Cairney, & Anderson, 2015; Jones & Smith, 2004; Mensah et al., 2015; Munkvold, Kjoller, Vestberg, Rosendahl, & Jakobsen, 2004; Watts‐Williams et al., 2019) and given the ubiquity of AMF in most agricultural soils, arable crops are far more likely to be mycorrhizal than nonmycorrhizal (Smith & Smith, 2011). As such, determining the conditions under which AMF positively influence crop nutrient uptake must remain a research priority. Plant and fungal genotype (Klironomos, 2003; Munkvold et al., 2004), the availability of mineral nutrients (Johnson, 2010; Johnson, Wilson, Wilson, Miller, & Bowker, 2015) and atmospheric conditions (Field et al., 2012) all mediate plant responses to AMF colonization.

Atmospheric CO_2_ concentrations ([CO_2_]) have increased rapidly because of anthropogenic activities since preindustrial times, from 280 ppm in 1750 to concentrations in excess of 400 ppm today (Meinshausen et al., 2011). Climate model projections suggest that atmospheric [CO_2_] will continue to rise, potentially reaching 800 ppm atmospheric [CO_2_] by the end of the century (Meinshausen et al., 2011) if steps to curb emissions are not taken. The ‘carbon fertilisation effect’ is responsible for increased rates of carbon fixation under elevated atmospheric [CO_2_] (hereafter eCO_2_), especially among C_3_ species in temperate zones (Ainsworth & Long, 2005; McGrath & Lobell, 2013; O'Leary et al., 2015) which include some of the world's most economically and socially important plants. As photosynthesis is not currently carbon‐limited at ambient atmospheric [CO_2_] (hereafter aCO_2_; Fitzgerald et al., 2016), plants grown at eCO_2_ generally show reduced photorespiratory losses and increased net photosynthetic rates. The extent to which increasing atmospheric [CO_2_] will impact crop–AMF associations remains unclear (Cotton, 2018). Given the key role of atmospheric [CO_2_] in regulating photosynthetic rate (van der Kooi, Reich, Low, Kok, & Tausz, 2016) and subsequent C metabolism, how AMF might ameliorate or accentuate any atmospheric [CO_2_]‐driven changes to crop growth and nutrition warrants further investigation.

As obligate symbionts, AMF are entirely reliant on their plant hosts for carbon (C) thus high atmospheric [CO_2_] could directly affect C allocation to mycorrhizas. Increased C acquisition by AMF has been demonstrated in a number of plant and fungal species when under eCO_2_ (Alberton, Kuyper, & Gorissen, 2005; Drigo et al., 2013; Field et al., 2012; Treseder, 2004). Furthermore, recent evidence even suggests that AMF carbon acquisition from host plants might directly increase rates of carbon fixation (Gavito, Jakobsen, Mikkelsen, & Mora, 2019), potentially by ameliorating end‐product inhibition of photosynthesis (Arp, 1991). Greater C acquisition by AMF may enable further hyphal proliferation through soil and thus increase their assimilation of mineral nutrients and subsequently increase transfer to host plants. However, whether this hypothetical positive feedback is realized in AMF–plant symbioses is not clearly supported by the available data (Cotton, 2018).

The nature and extent of atmospheric [CO_2_] effects on AMF are complex (Cotton, 2018). Increased plant N uptake via AMF under eCO_2_ has been demonstrated both in wild grasses, such as *Avena fatua* (Cheng et al., 2012) and in domesticated crop plants, including wheat *Triticum aestivum* L. (Zhu, Song, Liu, & Liu, 2016). In contrast, AMF‐mediated P uptake in vascular plants appears to be less affected by changes in atmospheric [CO_2_]. Mycorrhizal P uptake was not increased by eCO_2_ in *Pisum sativum* (Gavito, Bruhn, & Jakobsen, 2002; Gavito, Schweiger, & Jakobsen, 2003), *Medicago truncatula* or *Brachypodium distachyon* (Jakobsen et al., 2016). Similarly, *Plantago lanceolata* showed decreased ^33^P acquisition via AMF per unit of plant‐fixed carbon allocated to the fungi in eCO_2_ conditions (Field et al., 2012). Host plant genotype must also be considered when investigating the effect of environmental perturbation on symbiotic functioning between crops and AMF; intraspecific diversity is an important driver of variation in these interactions (Johnson, Martin, et al., 2015). As a result of intensive crop breeding to promote various economically important traits, modern crop cultivars vary in their receptiveness to colonization by AMF (Lehnert, Serfling, Enders, Friedt, & Ordon, 2017; Lehnert, Serfling, Friedt, & Ordon, 2018) and therefore potentially also vary in carbon‐for‐nutrient exchange between symbiotic partners in both aCO_2_ and eCO_2_ atmospheric conditions.

Here we address the critical research question, “How do eCO_2_ and plant host genotype affect carbon‐for‐nutrient exchange between wheat and arbuscular mycorrhizas?” Using ^15^N, ^33^P and ^14^C isotope tracers across three modern wheat (*T. aestivum* L.) cultivars, we determined (a) the extent to which AMF contribute to assimilation of N and P from soil, and (b) the extent to which wheat transfers C to extraradical mycelia of their fungal symbionts in three modern wheat (*T. aestivum* L.) cultivars at aCO_2_ (440 ppm) and eCO_2_ (800 ppm), to simulate the predicted increase in atmospheric [CO_2_] over the next 80 years (Meinshausen et al., 2011). Specifically, we tested the hypotheses that (a) AMF would acquire greater amounts of plant‐fixed C under future climate eCO_2_ scenarios, and (b) increased C allocation would increase transfer and assimilation of ^15^N and ^33^P tracers from the AMF to the plant across all cultivars tested.

## 2 MATERIALS AND METHODS

### 2.1 Wheat pregermination and AMF inoculation

Seeds of bread wheat (*T. aestivum* L., cv. ‘Avalon’, ‘Cadenza’, ‘Skyfall’; RAGT Seeds, Cambridgeshire, UK) were surface sterilized using Cl_2_ gas (Method S1) and incubated on moistened filter paper for 5 days to germinate. Avalon and Cadenza were selected as they are parent lines of a reference population currently used as a basis for improving European wheat germplasm (Ma et al., 2015), and Skyfall is currently among the United Kingdom's most commonly planted wheat cultivars. Healthy seedlings were selected and transferred to 1.5 L plant pots containing a 3:1 mix of agricultural top soil (collected on 7 December 2016 from Leeds University Farm; 53°52′30.1″N, 1°19′15.8″W) and heat‐sterilized (120 min at <120°C) soft sand (Figure S1).

To supplement the naturally occurring AMF inoculum in the field soil, an inoculum of the generalist mutualistic AMF species *Rhizophagus irregularis* (Kiers et al., 2011) was also added (Method S1). Homogenized inoculum was added to the sterilized sand immediately prior to mixing with the soil, with each pot receiving 10 ml of the inoculum. Spore density was quantified at 1,300 ± 100 spores per ml, such that each plant was inoculated with an additional 13,000 ± 1,000 *R. irregularis* spores.

### 2.2 Plant growth conditions

Plants were maintained in controlled environment growth cabinets (Snijder Labs) on a light cycle of 15 hr daytime (20°C and 70% humidity) and 9 hr night‐time (at 15°C and 70% humidity). Daytime PAR, supplied by LED lighting was 225 µmol m^−2^ s^−1^ at canopy level. CO_2_ concentrations were 440 and 800 ppm. Atmospheric [CO_2_] was monitored using a Vaisala sensor system (Vaisala), maintained throughout the addition of gaseous CO_2_. Plants were transferred between growth cabinets every 4 weeks to mitigate any cabinet effects. After 4 weeks, plants were given weekly doses of 40 ml of a low‐P preparation (containing 25% of the original P quantity) of Long Ashton solution (Smith, Johnston, & Cornforth, 1983), prepared using the nitrate formulation (Table S1). Plants were watered with tap water, as required.

### 2.3 ^33^P and ^15^N isotope tracing

Arbuscular mycorrhizal fungi‐mediated N and P assimilation was quantified using an approach adapted from Johnson, Leake, and Read (2001) using mesh‐walled cores, into which the ^33^P and ^15^N tracers were added. Briefly, each pot contained two mesh cores constructed from PVC tubing (length 80 mm, diameter 18 mm), with windows (approx. 50 mm × 12 mm) cut in each side (Figure S2). These windows and the bottom of each core were covered in a 20 µm nylon mesh which prevents root access but permits hyphal growth into the core contents. Nylon mesh was attached to PVC cores using Tensol^®^ adhesive (Bostik Ltd). Two of the cores were filled with the same soil and sand substrate as the bulk soil, plus 3 g/L crushed basalt (particle size <1 mm), to act as a fungal ‘bait’ (Quirk et al., 2012). Each pot also contained a third mesh‐windowed core, loosely packed with glass wool (Acros Organics) and then the top sealed with a SubaSeal^®^ (Perkin Elmer). This created an airtight septum through which gas sampling can be conducted with a hypodermic syringe, in order to measure belowground respiration throughout the course of the experiment.

To ensure only symbiotic fungal‐mediated tracer movement was measured, one of the mesh‐windowed soil cores in each pot was gently rotated immediately prior to isotope tracer additions, 10 weeks postplanting. This rotation severed the fungal connections between the plant and the core contents, preventing direct transfer of the isotope tracers to the host plants via extraradical mycorrhizal fungal mycelium. Core rotation was conducted every 48 hr until the end of the experiment. The second core in each pot remained static, thereby preserving the hyphal connections between the core contents and the host plant. After 10 weeks of growth, 100 µl labelling solution, containing 1 MBq ^33^P (as H_3_^33^PO_4_, specific activity = 111 TBq/mmol; Perkin Elmer) and 46.26 µg ^15^N (as >98 atom% ^15^NH_4_Cl; Sigma Aldrich) was introduced to each pot. Labelling solution was added via pierced capillary tubing running down the centre of the core to ensure even distribution of tracer within the core. In half of microcosms (*n* = 6 per cultivar), labelling solution was added to the static core, and in the remaining microcosms (*n* = 6 per cultivar), to the rotated core. Cores which did not receive tracer solution were given 100 µl autoclaved distilled H_2_O. By subtracting the quantity of isotope tracers detected in plants from pots with severed hyphal connections to the isotope core (rotated isotope core treatment) from those where the AMF mycelium remained intact (static isotope core treatment), we were able to account for movement of isotopes caused by dissolution and diffusion and/or alternative soil microbial nutrient cycling processes.

### 2.4 Plant‐to‐fungus carbon transfer

Two weeks after ^33^P and ^15^N tracer additions, plants were prepared for ^14^CO_2_ labelling, to allow movement of carbon from plant to fungus to be quantified. A 110 µl solution of NaH^14^CO_3_ (Perkin Elmer) containing 1.0175 MBq ^14^C (specific activity = 1.621 GBq/mmol) was added to a cuvette in each pot. The tops of all mesh‐windowed cores were sealed using gas‐tight rubber septa (SubaSeal) to minimize diffusion of ^14^CO_2_ into the cores. ^14^CO_2_ gas was liberated from the NaH^14^CO_3_ by addition of 10% lactic acid, generating a 1.0175 MBq pulse of ^14^CO_2_. Samples of 1 ml above‐ground gas and 1 ml below‐ground gas (via the glass wool‐filled core) were taken 1 hr after release of ^14^CO_2_ and every 4 hr thereafter to monitor the drawdown, respiration and flux of ^14^C through the plant–AMF network. Gas samples were injected into gas‐evacuated scintillation vials containing 10 ml Carbosorb^®^ (Perkin Elmer), a carbon‐trapping compound. To this, 10 ml Permafluor scintillation cocktail (Perkin Elmer) was added, and ^14^C content of each sample was quantified by liquid scintillation counting (Tricarb 3100TR scintillation counter; Perkin Elmer).

Pots were maintained under cabinet conditions until detection of maximum below‐ground ^14^C flux (20–22 hr after ^14^CO_2_ liberation) at which point 3 ml 2 M KOH was added to cuvettes within each microcosm to capture remaining gaseous ^14^CO_2_.

### 2.5 Harvest, sample preparation and analysis

All plant shoots, roots, bulk and core soil samples were separated, cleaned (roots only) and weighed before being immediately frozen and freeze‐dried (Scanvac Cool‐Safe freeze‐dryer; LaboGeneApS) within 24 hr. Shoot, root and soil samples were homogenized and subsamples of core and bulk soils were collected for quantification of hyphal length density. Subsections of roots were separated before freezing for quantification of mycorrhizal colonization using acidified ink (Vierheilig, Coughlan, Wyss, & Piche, 1998). Root colonization by AMF and the presence of arbuscules and vesicles was quantified by light microscopy using the protocol of McGonigle, Miller, Evans, Fairchild, and Swan (1990).

Plant phosphorus (nonradioactive) concentration was quantified by spectrophotometer assay following sulphuric acid digest. Sample P concentration was then calculated from a calibration curve constructed using known concentration of sodium dihydrogen orthophosphate. Briefly, plant root and shoot samples of known weight (30 ± 5 mg) were heated in a dry block heater (Grant Instruments) to 365°C in 1 ml 96% (v/v) sulphuric acid for 15 min. Once samples had cooled to 25°C, 0.25 ml 35% (v/v) hydrogen peroxide was added, at which point the samples turned colourless. Samples were again left to cool to 25°C. A 0.5 ml sample of this digest product was transferred to a 4 ml spectrophotometry cuvette, together with 0.2 ml 0.1 M l‐ascorbic acid (C_6_H_8_O_6_), 0.2 ml 3.44 M NaOH to neutralize acidity and 0.5 ml of a developer solution. The developer solution was prepared by dissolving 4.8 g of ammonium molybdate ((NH_4_)_6_Mo_7_O_24_.4H_2_O) and 0.1 g antimony potassium tartrate (C_6_H_4_O_7_SbK) in 250 ml 2 M H_2_SO_4_, which was then diluted to 500 ml with distilled water. The volume of sample in the cuvette was made up to 3.8 ml and samples were kept in the dark for 45 min, after which absorbance was measured at 882 nm using a Jenway 6300 spectrophotometer (Cole‐Palmer).

### 2.6 Quantification of carbon‐for‐nutrient exchange between plants and AMF symbionts

Shoot and root ^33^P content was quantified using aliquots of the digest product described above. About 1 ml aliquots of this digested product were mixed with 10 ml Emulsifier‐Safe (Perkin Elmer) and ^33^P was quantified by liquid scintillation counting. About 4 mg (±2 mg) of shoot and root tissue from all plants was weighed for analysis for ^15^N content by continuous‐flow mass spectrometry (PDZ Europa 2020 Isotope Ratio Mass Spectrometer coupled to PDZ ANCA GSL preparation unit). Data were collected as atom% ^15^N and %N using unlabelled plants for background detection. Quantification of plant ^15^N was calculated following the methods of Cameron, Leake, and Read (2006). About 15 mg (±2 mg) of shoot and root tissue, and (40 ± 5 mg) soil from static and rotated cores, and the bulk soil was subsampled for ^14^C quantification by liquid scintillation counting, following combustion using a sample oxidizer (Packard 307 Sample Oxidiser; Perkin‐Elmer).

Following the methods of Cameron, Johnson, Read, and Leake (2008), total C fixed by the plant and subsequently acquired by the fungus was calculated as a function of total CO_2_ volume in the labelling chamber and the proportion of the ^14^CO_2_ which was fixed by wheat plants over the labelling period (Figure S1). Comparing ^14^C quantities in static versus rotated cores for each pot allows calculation of C acquisition by the fungi, controlling for ^14^C detected due to root exudation or respiration, or alternative microbial carbon cycling processes.

### 2.7 Statistics

Statistical analyses were carried out using ‘R’ statistical software, version 3.4.3. (R Core Team, 2017), implemented within the RStudio graphical user interface (RStudio Team, 2015). Data were tested by two‐way ANOVA, where the cultivar and atmospheric [CO_2_] were used as predictor variables. Where ANOVA gave *p* < .05 for the main effects, Tukey post hoc tests were used to identify statistical differences between groups. Prior to running analyses, data were tested for normality using Shapiro–Wilk test and by visual inspection of residual plots. Where data did not pass assumptions of normality and homogeneity of variance, data were log_10_ transformed. Following results from Akaike information criterion (AIC) testing which showed better model fit, data were log‐transformed prior to statistical analysis.

## 3 RESULTS

### 3.1 Elevated [CO_2_] increases above‐ground wheat growth and frequency of intraradical mycorrhizal structures

**Figure 1:**
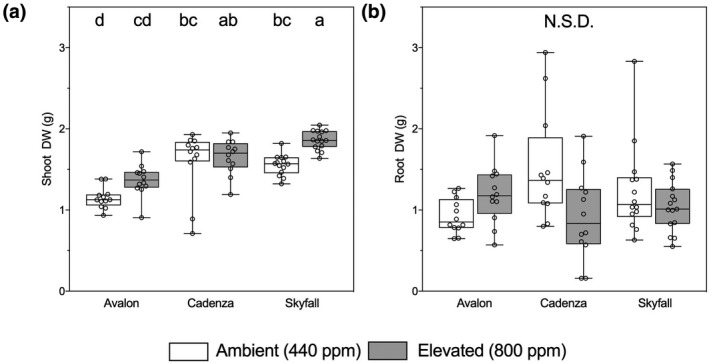
Root (a) and shoot (b) dry weight (g) of wheat (*Triticum aestivum* L., cv. Avalon, Cadenza, Skyfall) grown at ambient (440 ppm, white boxes) and elevated (800 ppm, grey boxes) CO_2_. Bars sharing letters are not significantly different, where *p* > .05 (ANOVA, Tukey post hoc test). Data were log_10_ transformed where data assumptions were not met. N.S.D., not significantly different

Plants grown under eCO_2_ (800 ppm) had on average 14% greater shoot biomass than those grown in aCO_2_ (440 ppm; Figure 1a; *F*_5,71_ = 16.33, *p* < .001), although among cultivars this response was only significant for Skyfall (Tukey test: *p* = .009). Mean cultivar shoot biomass ranged from 1.15 ± 0.04 g in Avalon grown at ambient [CO_2_] to 1.86 ± 0.30 g in Skyfall grown at eCO_2_. Root biomass did not respond to atmospheric [CO_2_] or cultivar (Figure 1b).

**Figure 2:**
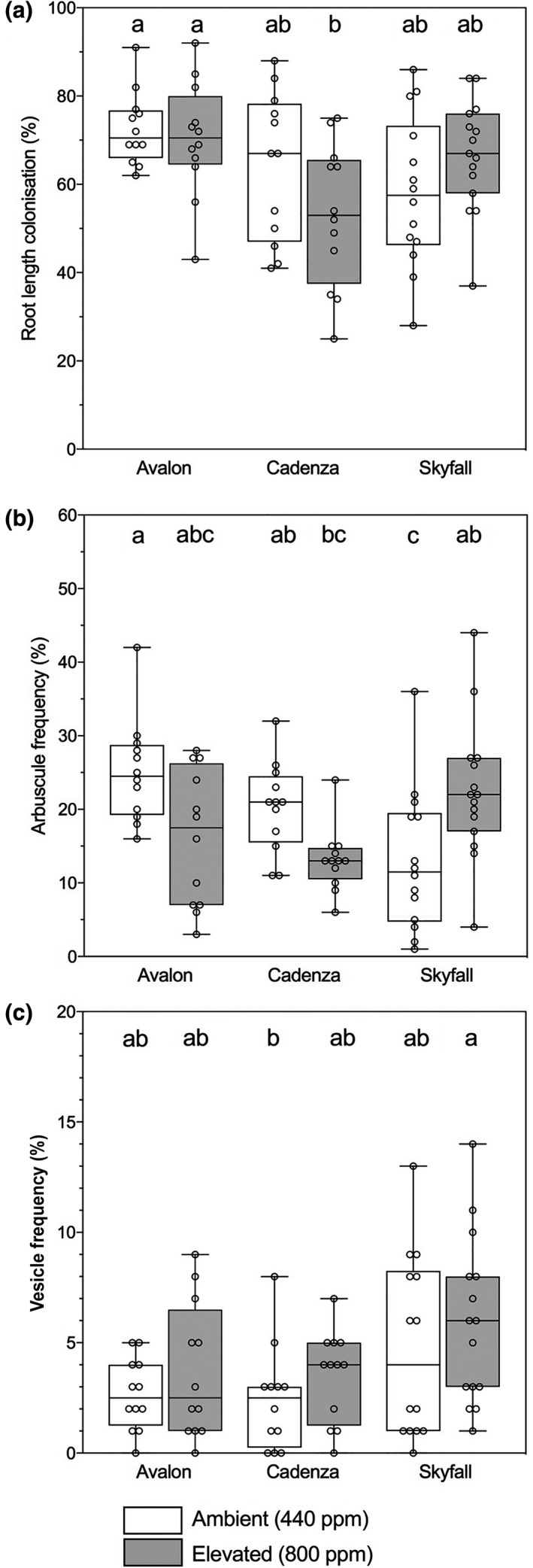
Root colonization (a), arbuscule frequency (b) and vesicle frequency (c) in roots of wheat (*Triticum aestivum* L., cv. Avalon, Cadenza, Skyfall) collected at harvest. Plants were grown at ambient (440 ppm, white boxes) and elevated (800 ppm, grey boxes) CO_2_. Bars sharing letters are not significantly different, where *p* > .05 (ANOVA, Tukey post hoc test). Data were log_10_ transformed where data assumptions were not met

All plants were colonized by AMF, with significant variation among cultivars in terms of per cent root length colonized (RLC; Figure 2a; *F*_2,74_ = 5.024, *p* < .01). Cadenza had significantly lower mean RLC (58.5 ± 3.5%) than Avalon (71.5 ± 2.2%), while mean Skyfall colonization (62.5 ± 2.8%) was not significantly different from either of the other cultivars (Tukey test: *p* > .05). The frequency of arbuscules was not affected by cultivar or atmospheric [CO_2_] although there was a significant interaction between these factors (Table S3), driven by reduced arbuscule frequency at eCO_2_ in Skyfall (Tukey: *p* < .001; Figure 2b). There is a trend towards greater vesicle abundance in wheat–AMF symbioses at 800 ppm than 440 ppm [CO_2_] across cultivars (Figure 2c), although this is not statistically significant.

### 3.2 Cultivar and aCO_2_ drive differences in plant P and mycorrhizal‐acquired ^33^P

**Figure 3:**
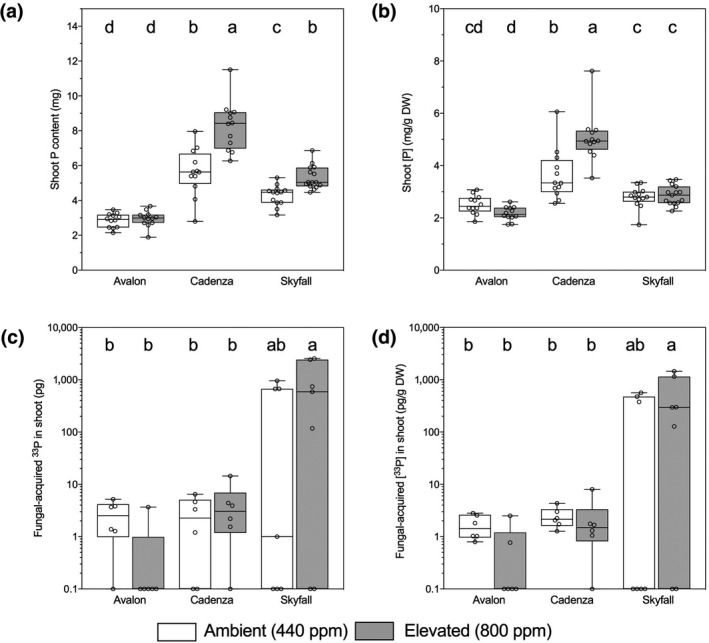
Shoot phosphorus (P) content (a) and concentration (b) of wheat (*Triticum aestivum* L., cv. Avalon, Cadenza, Skyfall) grown at ambient (440 ppm, white boxes) and elevated (800 ppm, grey boxes) CO_2_. Shoot content (c) and concentration (d) of fungal‐acquired ^33^P. Plants were grown at ambient (440 ppm, white boxes) and elevated (800 ppm, grey boxes) CO_2_. Bars sharing letters are not significantly different, where *p* > .05 (ANOVA, Tukey post hoc test). Data were log_10_ transformed where data assumptions were not met

There are strong effects of cultivar and atmospheric [CO_2_] effects on P content in shoots (*F*_5,70_ = 38.96, *p* < .001; Figure 3a). P content in Cadenza shoots was 196% greater than in Avalon shoots, and 137% higher than in Skyfall shoots. Similarly, P concentration in Cadenza shoots was 186% higher than in Avalon shoots, and 153% higher than in Skyfall shoots. Cadenza plants grown at eCO_2_ had the highest shoot P content and concentration of all cultivars for both atmospheric [CO_2_] treatments (Figure 3a,b). Root P content varied significantly by cultivar (*F*_2,73_ = 9.935, *p* < .001) but not CO_2_ concentration (*p* > .05). Combining data for CO_2_ treatments, Cadenza had the highest root P content (4.58 ± 0.49 mg), compared to Skyfall (3.33 ± 0.24 mg) and Avalon (2.34 ± 0.19 mg). Similarly, root P concentration was not affected by atmospheric [CO_2_], but varied by cultivar (*F*_2,73_ = 42.68, *p* < .001; Table S1). Avalon has significantly lower P concentration (2.08 ± 0.10 mg/g DW) in the roots than Skyfall (3.04 ± 0.12 mg/g DW) or Cadenza (3.86 ± 0.16 mg/g DW).

Plant assimilation of fungal‐acquired ^33^P tracer in cultivars Avalon and Cadenza (content and concentration; Figure 3c,d; Table S3) was reduced in eCO_2_ treatment, but slightly increased in Skyfall, although these trends were not statistically significant. There was high variability in ^33^P tracer uptake by Skyfall, requiring log_10_ transformation of the data to meet the assumptions of ANOVA. There were clear differences between cultivars in terms of ^33^P acquisition via mycorrhizas. Combining data from eCO_2_ and aCO_2_, Skyfall acquired 570 times more ^33^P tracer than Avalon and 225 times more than Cadenza (Figure 3c,d).

### 3.3 Cultivar‐specific differences in plant‐acquired N, but not mycorrhizal‐acquired ^15^N tracer

**Figure 4:**
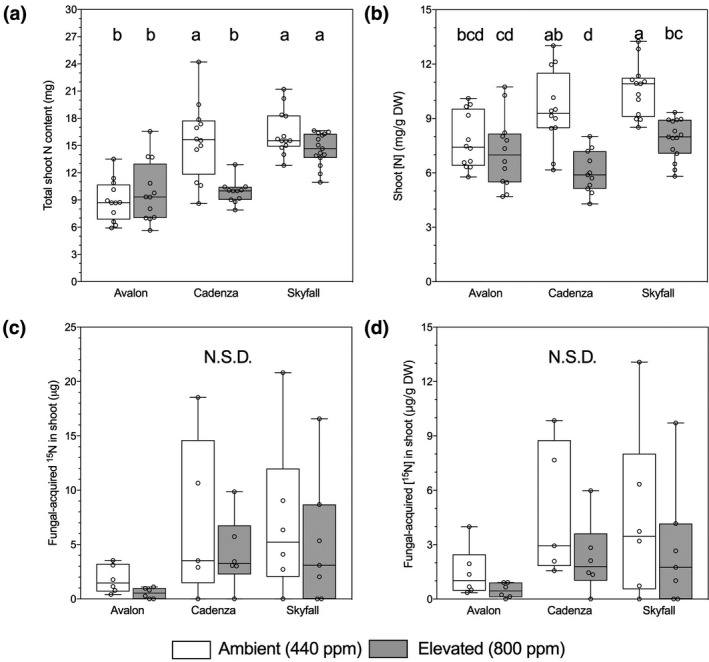
Shoot nitrogen (N) content (a) and concentration (b) of wheat (*Triticum aestivum* L., cv. Avalon, Cadenza, Skyfall) grown at ambient (white boxes) and elevated CO_2_ (black boxes). Shoot content (c) and concentration (d) of fungal‐acquired ^15^N. Plants were grown at ambient (440 ppm, white boxes) and elevated (800 ppm, grey boxes) CO_2_. Bars sharing letters are not significantly different, where *p* > .05 (ANOVA, Tukey post hoc test). Data were log_10_ transformed where data assumptions were not met. N.S.D., not significantly different

Elevated atmospheric [CO_2_] significantly decreased shoot N content in Cadenza (Tukey: *p* < .001; Figure 4a; Table S3) but not in Avalon and Skyfall. Cultivars also showed significant variation in shoot N content (Figure 4a; Table S3). Avalon shoots contained significantly lower N content than Cadenza (Tukey: *p* < .001) and Skyfall (Tukey: *p* < .001), while Cadenza and Skyfall did not significantly differ (*p* > .05). eCO_2_ also had a significant negative effect on shoot N concentration (*F*_1,74_ = 11.09, *p* = .001; Figure 4b), driven by large decreases in shoot N concentration in Cadenza (Tukey: *p* < .01) and Skyfall (Tukey: *p* < .01).

There were no significant differences among cultivars in total mycorrhizal‐acquired ^15^N tracer or concentration in shoots (Figure 4c,d), although there was a trend (not significant) across all three cultivars for greater ^15^N content in plants grown at aCO_2_ compared to those grown under the eCO_2_ treatment. ^15^N content and concentration of roots was not affected by atmospheric [CO_2_] or cultivar (Table S3).

#### 3.3.1 Cultivar‐specific carbon allocation to fungal partners

**Figure 5:**
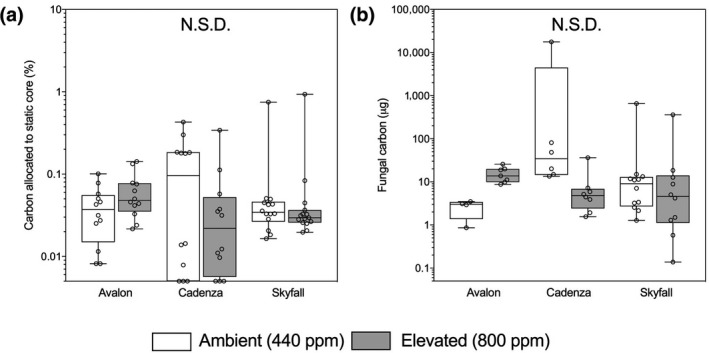
Total carbon transferred from wheat (*Triticum aestivum* L., cv. Avalon, Cadenza, Skyfall) to fungal mycelium during the course of ^14^C labelling experiment (a), and per cent of carbon fixed during the labelling period which was recovered in the static core at harvest (%) (b). Plants were grown at ambient (440 ppm, white boxes) and elevated (800 ppm, grey boxes). Bars sharing letters are not significantly different, where *p* > .05 (ANOVA, Tukey post hoc test). Data were log_10_ transformed where data assumptions were not met. N.S.D., not significantly different

All plants in both atmospheric [CO_2_] treatments transferred small amounts of carbon to the extraradical mycelium of their fungal symbionts (Figure 5a,b). The amounts were not significantly different between atmospheric [CO_2_] treatments in terms of per cent of carbon fixed during the labelling period allocated to the symbiotic fungi within the soil core (Figure 5a) or total amount of C transferred to extraradical fungal mycelium (ERM) within the pot (Figure 5b). However, there were trends suggestive of cultivar‐specific responses to eCO_2_ with per cent allocation of recent photosynthate and total amount of C transferred to fungal partners being greater at eCO_2_ than at aCO_2_ in cv. Avalon, lower in cv. Cadenza and unchanged in cv. Skyfall (Figure 5a,b). The hyphal length density in the bulk soil showed significant variation between cultivars (Tables S2 and S3; *F*_2,31_ = 15.79, *p* < .001); Avalon supported significantly less extraradical mycelium than Skyfall.

#### 3.3.2 Carbon‐for‐nutrient transfer between wheat and AMF

Carbon for nutrient transfer between plants and AMF was tested using Spearman's rank correlation coefficient (Figure S3). Overall, there was no correlation between fungal carbon acquisition and fungal transfer of ^33^P (*r*_s(34)_ = 0.025, *p* = .89) or ^15^N (*r*_s(34)_ = 0.067, *p* = .070) to host plants. There was also no correlation between the amounts of N and P transferred to host plants by AMF (*r*_s(34)_ = 0.18), *p* = .30). Spearman rank tests were also carried out on subset data, grouped by CO_2_ concentration, cultivar and factorial permutations of these. In no cases were there correlations between the nutrients transferred (data not shown).

## 4 DISCUSSION

Global atmospheric [CO_2_] is predicted to increase through the 21st century, and the effects of this change on crops remains uncertain. Maximizing the physiological benefits eCO_2_ may bring, such as increased photosynthetic rates (Ainsworth & Long, 2005), while minimizing deleterious effects such as reduced plant tissue nutrient concentration, presents a significant challenge. How far AMF may be useful in tackling this challenge, and their utility in wider agriculture generally, remains unclear (Cotton, 2018; Rillig et al., 2019; Ryan & Graham, 2018; Ryan, Graham, Morton, & Kirkegaard, 2019).

Significant variation in growth responses to colonization by AMF has previously been identified across cereal varieties (Hetrick, Wilson, & Cox, 1992; Lehnert et al., 2018; Watts‐Williams et al., 2019). Such genotypic differences in growth resulting from AMF symbioses are likely to be linked not only to the receptivity to fungal colonization, but also to the physiological function of the AMF associations, particularly the degree to which the fungal symbionts represent a carbon sink (Walder et al., 2012) and nutrient source (Watts‐Williams et al., 2019) for their host plants. The stoichiometry of the bidirectional exchange of plant carbon for fungal‐acquired nutrients characteristic of AM symbioses between cereals and AMF has, until now, remained unquantified.

### 4.1 Carbon outlay by wheat to AMF is unaffected by atmospheric [CO_2_]

In our experiments, plant biomass increased in eCO_2_ (Figure 1b). However, the C transferred to the extraradical mycelium, in terms of both total amounts, and per cent of recently fixed photosynthate, was not affected (Figure 5). This suggests that transfer of plant C to fungal symbionts in our experiments was not limited by availability of plant‐fixed C and that allocation of C to AMF by wheat is independent of its own C demand for growth. Plant photosynthates are used by AMF symbionts to build fungal structures both inside and outside their host plant cells. ERM is formed using carbon resources throughout the growth of both plant and fungal symbionts, and so the extent of fungal mycelium may be used to indicate the relative C allocation to fungal symbionts across a longer time period than the isotope tracing alone. We found no differences in ERM density between atmospheric [CO_2_] treatments (Tables S2 and S3) which supports our finding that atmospheric [CO_2_] does not affect wheat C allocation to fungal mycelium and that this is true across cultivars. However, there are strong effects of cultivar (Table S3) with greater C allocation to mycorrhizal fungi by Avalon compared to Cadenza or Skyfall plants over the course of the experiment.

Intracellular plant–fungal interfaces are formed, and degenerate, throughout the lifetime of the symbiosis. As such, the abundance of these structures, particularly those believed to serve fungal storage organs, may be used to infer relative plant carbon investment (Müller, Ngwene, Peiter, & George, 2017) over a longer period of time than the instantaneous measurements made through the isotope tracing approach used here. The frequency of vesicles, as fungal lipid stores, may be indicative of AMF carbon acquisition (Smith, Grace, & Smith, 2009). In our experiments, vesicle frequency did not differ between atmospheric [CO_2_] treatments (Figure 2c). Thus, it appears that there was no ‘carbon fertilisation effect’ of eCO_2_ for wheat‐associated AMF (Alberton et al., 2005). The lack of atmospheric [CO_2_] response in terms of AMF C acquisition observed in our experiments runs counter to the trends observed in meta‐analyses (Alberton et al., 2005; Treseder, 2004) and other experimental studies (Field et al., 2012). Intensive modern breeding programmes which have given rise to elite wheat cultivars such as those used in our experiments may be responsible for the lack of atmospheric [CO_2_] effect on AMF C acquisition. To maximize nutrient uptake efficiency in systems where fertilizer nutrients are applied in readily available forms (Good & Beatty, 2011), modern elite cereals are bred to have reduced root‐to‐shoot ratios compared to older cultivars (Siddique, Belford, & Tennant, 1990). Those cultivars with large root systems where nutrients are easily acquired could be viewed by breeders as C‐inefficient, as C allocated to below‐ground growth could be retained above‐ground. To this end, the allocation of C to mycorrhizas and ERM may have been inadvertently selected against in the breeding of modern cereal cultivars. Alternatively, the apparent lack of atmospheric [CO_2_] response observed here may be partly due to plant and fungal C allocation to AMF spores not being quantified in the present investigation; it is possible that under eCO_2_ the AMF produced greater number of spores than in aCO_2_. This would not have been quantified in our experiment given the relatively short ^14^CO_2_ labelling period, and might also account for a significant fraction of fungal C. In addition, AMF hyphal turnover is thought to be rapid (Staddon, Ramsey, Ostle, Ineson, & Fitter, 2003) and may represent a significant source of C input to soils (Godbold et al., 2006). Respiratory losses of hyphal‐derived C would not be quantifiable in our experimental approach. How atmospheric [CO_2_] affects hyphal turnover in AMF associated with crop plants remains to be determined.

The amounts of C allocated to AMF by the wheat cultivars in these experiments are similar to those recorded in comparable experiments with noncrop vascular plants (Field et al., 2012). However, only a small fraction of the total C fixed during the experimental period by the various wheat cultivars here was allocated to their fungal mycelium (Figure 5b), regardless of the availability of C in the atmosphere. Adding ^14^CO_2_ to an enclosed system, such as the labelling chamber in our experiments, inevitably leads to an increased CO_2_ concentration which would impact plant physiology. However, the addition of 1.1 MBq of ^14^CO_2_ to our labelling chambers increased the concentration of atmospheric [CO_2_] within the chambers by 1.24% in aCO_2_ and 0.36% in eCO_2_ treatments. This slight increase in atmospheric [CO_2_] is unlikely to have elicited a substantial physiological response in the plants used in our experiment. Given that our plants were only able to fix and assimilate ^14^CO_2_ for one photoperiod, it is likely that the amount of C measured by the isotope tracing was not reflective of total plant carbon allocation to symbiotic fungi across the life cycle of the plant; this warrants further investigation. Despite this, our experiment provides valuable insights into the allocation of recently fixed C to fungal symbionts of wheat during a period of rapid plant growth and high nutrient demand.

### 4.2 Cultivar‐specific wheat nutrient gains via mycorrhizas

All cultivars assimilated ^15^N and ^33^P via their mycorrhizal symbionts, with the amounts of each tracer varying according to the cultivar. Skyfall assimilated the most mycorrhizal‐acquired ^33^P tracer compared to cv. Avalon and Cadenza (Figure 4c,d). This pattern of nutrient gain from AMF is not reflected in the total nutrient content or concentration of plant tissues across cultivars (Figure 4a,b). Cadenza contains the most P, both fungal‐ and plant‐acquired, in its above‐ground tissues (Figure 4a,b) but it is cv. Skyfall that acquires the most ^33^P tracer via AMF symbionts. This pattern may be reflective of variation in nutrient acquisition strategies across the cultivars tested. Cadenza has the greatest P concentration of above‐ground tissues (Figure 4b), but lower AMF‐assimilated tracer content (Figure 4c) and concentration (Figure 4d) than other cultivars and thus appears to operate a more effective plant P assimilation pathway than cv. Skyfall, which appears to rely more heavily on the mycorrhizal pathway for nutrient acquisition (Smith, Smith, & Jakobsen, 2003; Smith et al., 2004). With the highest levels of AMF colonization (Figure 2a) and extraradical mycelial density (Table S2), but the lowest AMF contribution to ^33^P uptake (Figure 3c,d) and lowest above‐ground dry mass (Figure 1a), it appears that Avalon forms a less nutritionally mutualistic interaction with AMF than the other two culitvars tested, potentially resulting in suppression of growth. This observation is unlikely a result of the AMF exerting an excessive carbon “drain” given that cv. Avalon does not allocate more C to its AMF than the other cultivars tested (Figure 5), and that the percentage of C allocated to AMF by wheat is low compared to other plants (e.g. Field et al., 2012). Instead, it is possible that downregulation of plant phosphate transporters following AMF colonization may be partly responsible, and as a result, plant P uptake is reduced relative to the nonmycorrhizal counterpart (Li, Smith, Dickson, Holloway, & Smith, 2008). As we do not have nonmycorrhizal treatments to compare nutrient acquisition and growth in these cultivars against, it is not possible to determine whether AMF suppress growth of cv. Avalon but this certainly warrants further research.

Mycorrhiza‐mediated uptake of ^33^P and ^15^N tracers was not significantly influenced by atmospheric [CO_2_] in any of the cultivars tested (Figures 3c,d and 4c,d). This finding is counter to some modelling predictions (Bever, 2015) and some experimental data (Field et al., 2012) but is broadly in agreement with experiments conducted in *Pisum* (Gavito et al., 2002, 2003), *Brachypodium* and *Medicago* (Jakobsen et al., 2016) which also showed little effect of atmospheric [CO_2_] on AMF‐acquired plant nutrient assimilation. Increased total P content (i.e. plant‐ and mycorrhizal‐acquired) at eCO_2_ compared to aCO_2_ treatment in shoots of cvs. Skyfall and Cadenza is counter to the general observation that P, like N, is usually relatively diluted in plant tissues at eCO_2_ owing to increased plant biomass (Jakobsen et al., 2016). Increased P uptake at eCO_2_ is not unprecedented, however (Campbell & Sage, 2002), it may be due to changes in root morphology (Nie, Lu, Bell, Raut, & Pendall, 2013). Our ^33^P labelling suggests that the AMF were not responsible for this increased P uptake (Figure 4c,d).

Plant tissue N content and concentration may be reduced when plants are grown in eCO_2_ conditions, as a result of increasing plant biomass (Cotrufo, Ineson, & Scott, 1998; Hogy & Fangmeier, 2008; Taub, Miller, & Allen, 2008). This trend is apparent in cv. Cadenza and Skyfall plants in our experiments, although not in Avalon (Figure 3a,b). The phenomenon of reduced N content of arable crops has potentially serious implications for the nutritional quality of grain and grain‐based food products (Pleijel & Uddling, 2012). Here we show that symbiotic fungal contributions to plant N assimilation are similar across atmospheric [CO_2_] treatments and cultivars, suggesting that AMF‐acquired N in wheat may not increase as atmospheric [CO_2_] increases in the future. Alarmingly, a recent study suggests that although increased N fertilizer application is capable of increasing yields in elevated [CO_2_] atmospheres, it is incapable of maintaining N concentrations of plant tissues comparable to those achieved at aCO_2_ (Pleijel, Broberg, Hogy, & Uddling, 2019).

Our data support plant/cultivar identity as an important driver of mycorrhizal benefit to plant hosts (Field & Pressel, 2018; Klironomos, 2003; Walder & van der Heijden, 2015). However, our data do not support the notion that carbon for nutrient exchange between wheat and AMF are governed by a linear, ‘reciprocal rewards’ model of mutualism (Bever, 2015; Fellbaum et al., 2012; Kiers et al., 2011) as previously shown using Petri dish‐based microcosm systems (e.g. Kiers et al., 2011) or single AMF species inoculation (e.g. Fellbaum et al., 2012). In our systems, where plants were grown in a nonsterile soil‐based substrate, inoculation with *R. irregularis* from root organ cultures is likely to have resulted in a mixed intraradical AMF community of multiple species, probably dominated by *R. irregularis*. As such, our experimental strategy does not permit us to comment on the influence of fungal identity on wheat–AMF function beyond there being a mixed AMF community present here.

There was no correlation between assimilation of fungal‐acquired nutrients in wheat in our experimental microcosms and C transfer to fungal partners across all cultivars tested, regardless of the availability of CO_2_ for photosynthesis. The exchange of wheat carbon for fungal‐acquired nutrients observed here may be better explained by differences in plant–fungal receptiveness and compatibility (Walder & van der Heijden, 2015). Given that our experiments were conducted using a nonsterilised agricultural soil, there were likely additional interactions and feedbacks with soil microbes and fungi that may have influenced carbon‐for‐nutrient exchange dynamics with factors such as soil microbial community composition playing an influential role.

Inter‐ and intraspecific genetic variation in plants and their AMF symbionts has been identified as sources of functional diversity in arbuscular mycorrhizal symbiosis (Johnson, Martin, et al., 2015; Watts‐Williams et al., 2019). In complex systems such as these, disentangling the causes of variation in plant–fungal environment interactions can prove challenging. For instance, Watts‐Williams et al. (2019) demonstrated that the expression of a suite of assorted genes in *Sorghum bicolor* was dependent not only upon AM fungal identity but also *S. bicolor* cultivar identity. Furthermore, these effects were not seen exclusively in genes involved directly in symbiosis; there was altered expression in genes linked to defence response, stress response and maturation onset (Watts‐Williams et al., 2019). Further crop traits which are variable among cultivars, such as phosphorus use efficiency, may also determine the extent to which AMF are beneficial for host plants (Smith & Smith, 2011). Perhaps surprisingly, root architecture traits may have limited effects on a plant's nutritional and growth response to mycorrhization (Maherali, 2014). Inter‐ and intraspecific functional diversity is also present in AMF species (Jones & Smith, 2004; Mensah et al., 2015; Munkvold et al., 2004; Watts‐Williams et al., 2019). By using unsterilized soil in our experiment, our experimental plants are likely to have been colonized by a mixed community of AMF, where the relative contributions of individual species or isolates cannot be ascertained. As AMF community structure is understood to impact symbiotic function (Frew, 2019; Smith et al., 2004; van der Heijden et al., 1998), this is of great potential agronomic interest. Understanding the role of genetic variability in plant–fungal interactions to the point where it can begin to help informing agriculture will likely prove to be a substantial, but ultimately worthwhile, undertaking (Johnson, Martin, et al., 2015). Metagenomic techniques should identify species and intraspecific diversity of the AMF present within field‐crop plant roots, combined with functional studies to determine the role these fungi play in crop nutrient uptake or other non‐nutritional beneficial roles. As illustrated by the present investigation, further factors to consider include the effects of abiotic factors on AMF community structure and diversity. Recent field‐scale atmospheric [CO_2_] manipulation has shown how CO_2_ enrichment can affect AMF community composition (Cotton, Fitter, Miller, Dumbrell, & Helgason, 2015; Maček et al., 2019). How these atmospheric [CO_2_]‐driven community changes might influence the stoichiometry of carbon‐for‐nutrient exchange between symbionts in the field remains to be determined (Cotton, 2018).

### 4.3 Future perspectives

Our results, and those of other studies investigating mycorrhizal responses to eCO_2,_ must be contextualized with the likelihood that climate change will encompass shifts in multiple abiotic variables. Factors such as N deposition, warming and drought are at least as important an influence on AMF as atmospheric [CO_2_] (Kivlin, Emery, & Rudgers, 2013). Our data demonstrate that AMF will continue to provide N and P nutrition to their plant hosts under eCO_2_ and that there is no evidence for significant C drain from the fungi. Whether these trends are seen following simultaneous perturbations of temperature, water availability and N deposition in crop plants is not clear, as experimental testing of such scenarios is lacking.

While AMF may not prove to be the silver bullet, ‘sustainable saviours’ for agricultural intensification (Thirkell et al., 2017), our experiments have demonstrated that AMF do have the potential to contribute to cereal nutrient assimilation. As such, AMF could have an important role to play in reducing application of N‐ and P‐based fertilizers as part of a wider strategy for sustainable soil management. We echo calls for further field scale experimentation of the function of AMF in crop plants to determine what role, nutritional or otherwise, AMF might be playing in crop growth in situ (Lekberg & Helgason, 2018; Rillig et al., 2019). To date, very little work has been carried out on crop breeding to optimize mycorrhizal benefit. Given the potential influence of AMF on plant nutrient uptake and growth (Klironomos, 2003) and their ubiquity in farm systems (Oehl, Laczko, Oberholzer, Jansa, & Egli, 2017; Sale et al., 2015) it appears remiss that AMF should not be considered in breeding programmes. Recent steps have been taken to investigate the genetic basis for mycorrhizal colonization (Lehnert et al., 2017) as well as mycorrhizal “benefit” and drought response in wheat (Lehnert et al., 2018), while similar efforts in other crop species have been in progress for several years (De Vita et al., 2018; Galvan et al., 2011; Kaeppler et al., 2000). Better understanding of the mechanisms underlying plant–microbial interactions remains important in the future‐proofing and sustainable intensification of agriculture.
