# Supplementary material for: Carbon for nutrient exchange between arbuscular mycorrhizal fungi and wheat varies according to cultivar and changes in atmospheric carbon dioxide concentration
Source: Glob Chang Biol. 2019 Oct 23;26(3):1725–38. doi: 10.1111/gcb.14851 (PMC7079082; doi:10.1111/gcb.14851)
Supplement: Supplementary file 1 [file GCB-26-1725-s001.docx]

*Global Change Biology* Supporting Information

# **Carbon-for-nutrient exchange between arbuscular mycorrhizal fungi and wheat varies according to cultivar and changes in atmospheric CO_2_ concentration**

Authors: Tom J. Thirkell; Daria Pastok, Katie J. Field*.

Centre for Plant Sciences, School of Biology, Faculty of Biological Sciences, University of Leeds, Leeds, LS2 9JT, UK.

* Corresponding author – email: [k.field@leeds.ac.uk](mailto:k.field@leeds.ac.uk)

15N

^15^N

^33^P

^14^CO_2_

**Figure S1.** Schematic diagram of experimental setup for isotopic labelling. Wheat (*Triticum aestivum* L. cv. Avalon, Cadenza and Skyfall were grown in 1.5 L pots containing a 1:1 mix of sand and top soil, inoculated with ≈ 15,000 spores of the arbuscular mycorrhizal fungus (AMF) *Rhizophagus irregularis*. Plants were grown at ambient (440 ppm) or elevated (800 ppm) CO_2_. To quantify wheat nitrogen (N) and phosphorus (P) acquisition via AMF, a solution containing ^15^N and ^33^P was added to a mesh-walled core in each pot, into which AMF hyphae but not roots could grow. In control microcosms, labelling cores were rotated every 48 hours to sever hyphae and prevent AMF ingrowth. To quantify plant allocation of carbon to AMF, microcosms were enclosed in polypropylene bags to create an airtight headspace and ^14^CO_2_ was introduced by adding lactic acid to NaH^14^CO_3_.


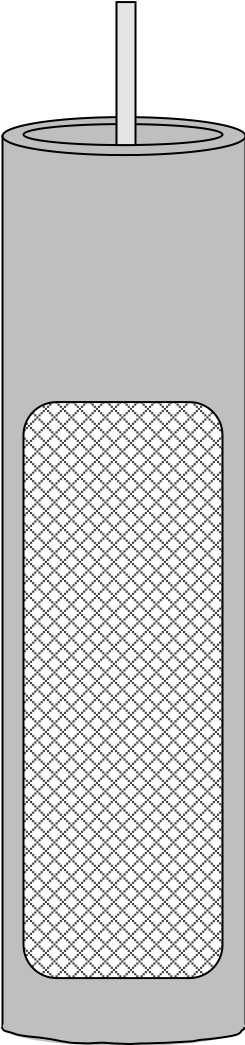


**Figure S2.** Schematic diagram of mesh-walled core into which isotope label solution was added. PVC tubing measuring 100 mm x 22 mm (internal diameter 18 mm) had windows cut in opposite sides of the lower half so that ≈ 50% of the area was open. These windows, and the bottom of the core were covered in 20 µm nylon mesh which allows the ingrowth of arbuscular mycorrhizal hyphae, but not roots. Mesh was fixed to PVC cores using Tensol acrylic cement adhesive. To allow addition of aqueous ^33^P/^15^N labelling solution, a silicone rubber capillary tube was perforated along its length, then fixed to the bottom of the core using silicone sealant.

**Figure S3.** Carbon for nutrient transfer between plants and AMF **(a)** and fungal-acquired ^15^N in plant tissue vs fungal-acquired ^33^P in plant tissue **(b)**. Correlation tested using Spearman’s rank correlation coefficient.

**Table S1.** ‘Long Ashton’ nutrient solution protocol detailing quantities of nutrients provided to *Triticum aestivum* plants through the experiment. Plants were given 40 mL of this solution weekly from 4 weeks after germination until harvest.

| Compound | Concentration in solution (mM) |
| --- | --- |
| KNO_3_ | 4 |
| Ca(NO_3_)_2_.4H_2_O | 4 |
| NaH_2_PO_4._2H_2_O | 0.2925 |
| MgSO_4_.7H_2_O | 1.5 |
| FeNaEDTA | 0.09 |
| MnSO_4_.4H_2_O | 0.01 |
| ZnSO_4_7H_2_O | 0.001 |
| CuSO_4._5H_2_O | 0.001 |
| H_3_BO_3_ | 0.05 |
| NaMoO_4_.2H_2_O | 0.0005 |
| NaCl | 0.1 |

|  | Avalon | | Cadenza | | Skyfall | |
| --- | --- | --- | --- | --- | --- | --- |
|  | 440 ppm | 800 ppm | 440 ppm | 800 ppm | 440 ppm | 800 ppm |
| Hyphal length density  (cm g^-1^ soil) | 55.75 ^c^  ± 3.63 | 50.50 ^bc^  ± 8.86 | 35.33 ^abc^  ± 5.20 | 38.60 ^abc^  ± 4.13 | 35.09 ^ab^  ± 1.04 | 20.80 ^a^  ± 2.06 |
| Root N content  (mg) | 5.75 ^a^  ± 0.48 | 6.19 ^a^  ± 0.70 | 10.67 ^b^  ± 0.63 | 6.52 ^a^  ± 1.14 | 5.47 ^a^  ± 0.51 | 6.97 ^a^  ± 0.67 |
| Root N concentration (mg g^-1^ DW) | 6.23 ^ab^  ± 0.39 | 4.88 ^a^  ± 0.51 | 8.17 ^b^  ± 0.54 | 7.94 ^b^  ± 0.63 | 4.65 ^a^  ± 0.26 | 6.66 ^b^  ± 0.28 |
| Root P content  (mg) | 1.88 ^a^  ± 0.19 | 2.79 ^ab^  ± 0.28 | 5.34 ^c^  ± 0.58 | 3.83 ^bc^  ± 0.74 | 3.44 ^ab^  ± 0.41 | 3.24 ^ab^  ± 1.10 |
| Root P concentration  mg g^-1^ DW | 2.02 ^a^  ± 0.14 | 2.15 ^a^  ± 0.14 | 3.64 ^bc^  ± 0.24 | 4.10 ^c^  ± 0.21 | 2.94 ^b^  ± 0.18 | 3.14 ^b^  ± 0.17 |
| Fungal-acquired root ^15^N content (µg) | 1.17 ^a^  ± 0.32 | 0 ^a^  ± 0 | 7.19 ^a^  ± 2.63 | 3.92 ^a^  ± 1.85 | 2.45 ^a^  ± 1.23 | 2.07 ^a^  ± 1.39 |
| Fungal-acquired root ^15^N concentration  (µg g^-1^ DW) | 1.66 ^a^  ± 0.40 | 0 ^a^  ± 0 | 5.79 ^a^  ± 2.23 | 4.33 ^a^  ± 3.23 | 1.47 ^a^  ± 1.18 | 2.18 ^a^  ± 1.22 |
| Fungal-acquired root ^33^P content (pg) | 0.35 ^a^  ± 0.13 | 0.89 ^a^  ± 0.37 | 2.464 ^a^  ± 0.71 | 2.20 ^a^  ± 1.05 | 160.00 ^b^  ± 44.60 | 54.22 ^ab^  ± 36.00 |
| Fungal-acquired root ^33^P concentration  (pg g^-1^ DW) | 0.71 ^a^  ± 0.21 | 0.625 ^a^  ± 0.40 | 1.83 ^a^  ± 0.55 | 2.51 ^a^  ± 1.11 | 105.22 ^b^  ± 45.91 | 56.91 ^ab^  ± 29.49 |

**Table S2.** Fungal and plant growth parameters, including fungal-acquired ^15^N and ^33^P for 3 cultivars of wheat (*Triticum aestivum* L. cv. Avalon, Cadenza, Skyfall). Data shown are means ± SEM, cells within a row sharing letters do not significantly differ.

| Variable | df | *F* value | | |
| --- | --- | --- | --- | --- |
|  |  | CO_2_ | Cultivar | CO_2_ x cultivar |
| DW shoot | 2,71 | 17.1428*** | 30.4823*** | 1.7677 |
| DW root | 2,71 | 1.0068 | 0.0379 | 6.5711** |
| Root length colonised | 2,71 | 0.1010 | 5.2727** | 3.3084* |
| Arbuscule frequency | 2,71 | 0.2144 | 1.1188 | 11.468*** |
| Vesicle frequency | 2,71 | 3.2329 | 3.6359* | 0.87573 |
| Shoot N content | 2,69 | 7.5982** | 37.0704*** | 7.8771*** |
| Shoot N concentration | 2,69 | 35.2289*** | 9.5838*** | 4.6770* |
| Shoot P content | 2,70 | 33.3022*** | 141.8793*** | 8.7523 |
| Shoot P concentration | 2,70 | 4.7102* | 81.1811*** | 13.8527*** |
| Shoot ^15^N content | 2,30 | 1.4506 | 4.5458* | 0.0405 |
| Shoot ^15^N concentration | 2,30 | 3.2578 | 3.8334* | 0.0801 |
| Shoot ^33^P content | 2,32 | 1.4724 | 7.8565** | 1.2586 |
| Shoot ^33^P concentration | 2,32 | 1.1238 | 7.5367** | 0.9743 |
| Fungal C | 2,71 | 0.3501 | 1.2326 | 2.8573 |
| % Carbon allocation | 2,71 | 0.0134 | 1.4815 | 1.1386 |
| Hyphal length density | 2,31 | 3.96 | 15.79*** | 2.72 |

**Table S3.** ANOVA *F* statistics for main treatment effects (CO_2_ and cultivar) and treatment interactions for *Triticum aestivum* L., cv. Avalon, Cadenza and Skyfall, produced from two-way ANOVA. ‘ ‘ = not significant; ‘*’ = p < 0.05; ‘**’ = p < 0.01; ‘***’ = p < 0.001.

**Methods S1**

*Wheat pre-germination and AMF inoculation*

Seeds of three elite, modern wheat (*Triticum aestivum* L.) cultivars ‘Skyfall’, ‘Cadenza’ and ‘Avalon’ (supplied by R.A.G.T. Seeds, Grange Road, Ickleton, Essex, UK) were surface-sterilised by 3 hours’ exposure to chlorine gas, liberated from a sodium hypochlorite solution (NaClO) using 10 % (v/v) HCl. Seeds were then transferred to wetted cellulose filter papers (Whatman No 1., Thomson Fisher Scientific) in 90 mm Petri dishes, and incubated for 5 days at 20 °C to germinate. The field from which soil was taken was being used for a cover crop of beans (*Vicia faba* L.), following a crop of winter wheat (*Triticum aestivum* L.) the previous year. Soil was taken from between 5 and 15 cm below the surface. After collection, soil was passed through a 2 mm sieve to remove stones and larger organic debris.

Inoculum was prepared by growing T-DNA transformed carrot (*Daucus carota* L.) root on MSR media (Declerk et al., 2005). Cultures were incubated in the dark at 22 °C. To allow inoculum application, plated media was briefly pulverised in a counter-top blender (Morphy Richards, Mexborough, UK) for around 30 seconds, until root fragments had been reduced to c. 3 mm in length. AMF inoculum was between 9-12 months old when added to plants. For each 100 mL of inoculum, 25 mL distilled water is added, to reduce viscosity and to allow spores and root fragments to be mixed homogenously though the growth medium.

To minimise evaporative water loss, algal growth and weed germination in the soil, a 1 cm deep layer of 3 mm diameter polypropylene beads (Northern Polymers and Plastics Ltd, Crewe, UK) was added to the surface of the pots 2 weeks after planting. Plants were grown in blocks owing to growth chamber growth restrictions. Skyfall was planted on 16/01/2017 and harvested on 28/03/2017; Avalon was planted on 20/04/2017 and harvested on 05/07/2017; Cadenza was planted on 15/06/2017 and harvested on 24/08/2017.

*Root staining*

Briefly, roots were cleared in 10 % KOH at 70 ˚C for 40 minutes before staining for 20 minutes at 20˚ C in a solution of black ink (Pelikan ‘Brilliant Black’, Pelikan Vertriebsgesellschaft & Co. mbH, KG, Hanover, Germany), acetic acid and distilled H_2_O in the ratio 1:1:18.

*^14^C isotope quantification*

^14^CO_2_ was liberated by adding 2 mL 10 % (v/v) lactic acid to the NaH^14^CO_3_. Pots were enclosed in gas-tight polythene bags (50 µm polythene (Polybags Ltd, Greenford, UK)), sealed with insulation tape at the top and around the rim of the pot.

Fungal C acquisition was quantified using equations 1-3:

(1) *FC* = (*SR*/ *SA*) m_a_

where *FC* = fungal carbon acquisition during labelling period, *SR* = sample radioactivity (Bq), *SA* = specific activity of ^14^C source (Bq mol^-1^) and m_a_ = molecular weight of ^14^C (14).

(2) *FC* = ((SR/SA) m_a_) + (*PC* x *MC*)

where *PC* = proportion of ^14^C supplied is present in the sample, *MC* = mass of carbon in the labelling clamber (g) (derived from the ideal gas law, detailed in equation 3).

(3) *mco* = *mmco (PVco / RT)* $\therefore$ *MC = mco x 0.279292*

where *mco* = mass of CO_2_ (g), *mmco* = molecular mass of CO_2_ (44.01 g mol^-1^), *P* = pressure (kPa); *Vco* = volume of CO_2_ in the chamber, *MC* = mass of unlabelled C in the labelling chamber (g), *R* = universal gas constant (J K^-1^ mol^-1^), *T*= absolute temperature (K), multiplied by the proportion of CO_2_ that is C, on a mass fraction basis, i.e. 27.292%.

Further samples of known weight of plant tissue (30 mg ± 5mg) and soils (100 mg ± 20 mg) were taken for acid digestion to allow quantification of P and ^33^P content. Tissue ^33^P content was calculated using formula 4, from Cameron et al. (2006):

(4) *m^33^P = ((DPM/60/SA) MW) DF.*

Where *m^33^P* = mass of ^33^P, *DPM* = disintegrations per minute, *SA* = specific activity of the ^33^P source (Bq mmol^-1^), *MW* = molecular weight of P, and *DF* = dilution factor.
